# Bloodless Reduction of Congenital Hip Dislocation

**Published:** 1902-02

**Authors:** 


### Bloodless Reduction of Congenital Hip Dislocation.

Dr. Walter G. Stern, in Pediatrics, states that congenital hip dislocations are even more frequent than club foot. Since 1890 four European operators alone have handled 1300 cases. After describing at some length and in detail the symptoms of the condition and the method of bloodless reduction as practiced by Lorenz, of Vienna, Dr. Stern concludes his article as follows: "The superiority of the bloodless reduction of Lorenz over the older bloody method lies in the fact that the dangers of sepsis, hemorrhage, shock and, above all, the subsequent ankylosis which is such a frequent termination of the open operation are avoided. The consent for the bloodless operation can be more easily obtained, the results are equally if not more certain than in the opposing operation, while the motion function and power of the joint are restored to almost their normal conditions." R.

